# Biological Control of Leaf Blight Disease Caused by *Pestalotiopsis maculans* and Growth Promotion of *Quercus acutissima* Carruth Container Seedlings Using *Bacillus velezensis* CE 100

**DOI:** 10.3390/ijms222011296

**Published:** 2021-10-19

**Authors:** Sang-Jae Won, Jae-Hyun Moon, Henry B. Ajuna, Su-In Choi, Chaw Ei Htwe Maung, Sangtae Lee, Young Sang Ahn

**Affiliations:** 1 Department of Forest Resources, College of Agriculture and Life Sciences, Chonnam National University, Gwangju 61186, Korea; lazyno@naver.com (S.-J.W.); mjh132577@naver.com (J.-H.M.); ajunahenry@mmu.ac.ug (H.B.A.); suin917@naver.com (S.-I.C.); 2 Division of Agricultural and Biological Chemistry, Institute of Environmentally Friendly Agriculture, College of Agriculture and Life Sciences, Chonnam National University, Gwangju 61186, Korea; chaweihtwemaung@gmail.com; 3 Forest Technology and Management Research Center, National Institute of Forest Science, Pocheon-si 11186, Korea; lst9953@korea.kr

**Keywords:** forest seedling production, antagonistic bacteria, lytic enzymes, leaf blight, auxin, root development, nutrient uptake

## Abstract

Leaf blight disease caused by *Pestalotiopsis* *maculans* lead to deleterious losses in the quality of forest container seedlings. The use of plant growth-promoting bacteria provides a promising strategy to simultaneously control diseases and enhance forest seedling production. This study investigated the biocontrol of leaf blight disease and growth promotion potential of *Bacillus velezensis* CE 100 in *Quercus acutissima* Carruth seedlings. *B. velezensis* CE 100 produced cell wall degrading enzymes, such as chitinase, β-l,3-glucanase, and protease, which caused cell wall lysis and hyphae deformation of *P. maculans*, leading to mycelial growth inhibition by 54.94%. Inoculation of *B. velezensis* CE 100 suppressed *P. maculans* infection and increased seedling survival rate by 1.6-fold and 1.3-fold compared to chemical fertilizer and control, respectively. In addition, *B*. *velezensis* CE 100 produced indole-3-acetic acid, which improved root development and nutrient uptake compared to chemical fertilizer and control. Especially, inoculation with *B. velezensis* CE 100 increased the total nitrogen content of *Q. acutissima* seedlings, improved the chlorophyll index in the leaves, and increased seedling biomass by 1.3-fold and 2.2-fold compared to chemical fertilizer and control, respectively. Thus, *B. velezensis* CE 100 could be applied in the eco-friendly production of high-quality forest seedlings.

## 1. Introduction

*Quercus* spp. (Oaks) are large deciduous trees with a broad spreading crown that are widely grown across Asia, Europe, and North America for their high commercial and environmental value [1,2,3]. Oaks have been used for various purposes including fuelwood and timber production [3,4], bed logs for mushroom cultivation [5], tannin extraction and bio-oil production [2,6], and acorn production [1]. They also have low carbon monoxide peak yield for safe fuel-wood production with reduced risk of gaseous poisoning [4] and are more resistant fire out-break [7]. Oaks are valuable forest trees with good adaptability to dry soil conditions via their deep-penetrating root systems and have the ability to conserve water because of their small stomata [8]. Thus, oaks are favorable trees for reforestation programs due to their high economic value and environmental adaptability. Accordingly, *Quercus acutissima* Carruth has recently been among the most planted forest tree species in Korea, contributing approximately 32.2% of broad-leaved trees and 15.3% of total forest coverage [3,9].

Successful afforestation requires strong and healthy forest seedlings with a well-developed root system for rapid establishment upon out planting, vigorous growth, and high survival under competitive conditions with weeds [10,11,12,13]. Traditionally, most oak seedlings have been produced by the bare-root seedling method in order to reduce the production costs [12]. However, bare-root seedlings easily lose their roots during lifting, storage, and transportation from the nursery to the field [12,13]. This increases transplant shock, and reduces the competitiveness with weeds for moisture and mineral nutrient absorption, thereby slowing down seedling growth and lowering the survival rate after transplantation [10,12,13]. In recent reforestation projects, container seedlings have been proposed as an alternative to bare-root seedlings for successful afforestation [11,12]. Container seedlings usually have a lower shoot to root ratio, which confers a higher chance of drought avoidance by maximizing water and nutrient absorption [13]. Moreover, the root systems of container seedlings are surrounded by growth media, which protects them from damage during transplantation [12,13]. Container seedlings have a well-developed root system, with more first-order lateral root growth and fibrous roots that contribute to increased nutrient and water absorption capacity after transplantation, which enhances the growth and survival of the seedlings [11,12,13,14]. Thus, to achieve the afforestation targets, many studies on the production of oak seedling have emphasized the production of high-quality container seedlings with a well-developed root system for successful competition and vigorous growth [11,12,14].

**Figure 1:**
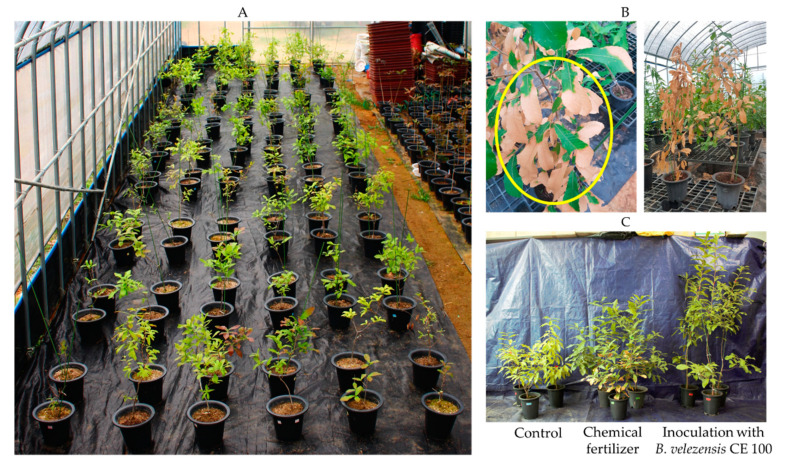
Study area in an experimental greenhouse (**A**), symptoms of *Pestalotiopsis maculans* infection in *Quercus acutissima* (**B**) and *Q. acutissima* seedling growth under different treatments (**C**).

However, in the production of container seedlings, nutrients, such as nitrogen and phosphorus can easily leach from the growth media and thus requires frequent use of chemical fertilizers to prevent nutritional imbalances [15]. The use of chemical nitrogen fertilizers to manage nutrient imbalances in container seedlings increases the density of phytopathogenic fungi in the growth medium [16]. In addition, the mass cultivation of single species seedlings at high density could potentially increase the transmissibility of fungal diseases across the nursey [17,18]. In this study, leaf blight incidence and mortality of *Q. acutissima* container seedlings were observed in an experimental greenhouse (Figure 1). After isolation of potential phytopathogens from leaves of dead seedlings, *Pestalotiopsis maculans* was identified as the source of pathogenicity. Previously, three *Quercus* species: *Q. petreae, Q. suber*, and *Q. ilex*, from Europe were imported to Fuyang, China [19]. One-year-old seedlings at a height range of 1 to1.5 m tall were planted using the bare root method and tested for susceptibility against major fungal pathogens. The symptoms of *Pestalotiopsis* infection, such as necrotic spots on the leaves, were only noted in *Q. petraea* [19]. *Pestalotiopsis* spp. have also been reported to cause a wide range of blight diseases to various container-grown seedlings and trees, such as leaf spot disease in Japanese persimmon (*Diospyros kaki* Thunb) [20], twig blight in hazelnut (*Corylus avellana* L.) [21], twig blight bayberry (*Myrica rubra* Sieb. et Zucc) [22], grey blight in som (*Persea bombycina* Kost) [23], and needle blight in pine (*Pinus* spp.) [24]. The symptoms of *Pestalotiopsis* blight starts as small spots, which under severe conditions could develop into necrotic lesion, total leaf chlorosis, dieback, loss of yield, and total plant mortality [20,21,22]. According to Yasuda et al. [20], *Pestalotiopsis* infection initially causes browning, starting from the tip (and/or edge) of the leaf, and eventually results in leaf necrosis. In severe cases, necrotic lesions occur in more than half of the leaf, and eventually spread throughout the leaf [20]. The infected leaves gradually turn brown as the disease spreads to other leaves and branches. In severe cases, the disease symptoms spread throughout the seedlings and cause wilting [22]. *Pestalotiopsis* blight of forest trees has been reported in different regions including China [19,22], Japan [20], Europe [21], and India [23].

Thus, it is vital to control *Pestalotiopsis* infections in forest seedlings, in order to produce high-quality seedlings for successful afforestation. Consequently, nursery operators frequently use chemical fungicides to efficiently control phytopathogenic fungi in the production of forest container seedlings [17]. For example, several systemic fungicides, such as Bavistin (Carbendazim), Topsin-M (Thiophanate-methyl), Mancozeb (ethylene-bis-dithiocarbamate), as well as copper oxychloride at high concentrations, have been reported for potential control of *Pestalotiopsis* blight [25]. Carbendazim and thiophanate-methyl (which interferes with mitotic cell process by binding on tubulin to suppress microtubule assembly) as well as mancozeb and copper oxychloride (which disrupts a wide range (multisite activity) of fungal cell cytoplasmic and mitochondrial activities that are catalyzed by enzymes containing sulphydryl groups), have potential to inhibit non-target microorganisms, including beneficial bacteria and streptomycetes [25]. However, frequent application of chemical fungicides could potentially cause fungicide resistance [26], pollutes the environment, and negatively affects human health [27]. Recently, interest in the production of forest seedlings using eco-friendly technologies to replace the use of chemical products has increased and several studies have provided evidence about the control of plant disease using plant growth-promoting bacteria (PGPB) [28,29,30,31,32,33,34]. Specifically, *Bacillus* spp. have demonstrated a wide range biocontrol potential against plant fungal diseases through the production of various secondary metabolites [28,29,33,34]. For instance, *Bacillus velezensis* was previously reported to produce antifungal cyclic tetrapeptide [33]. However, such antibiotic compounds are often produced in low concentrations, which makes their efficacy under field conditions insufficient [30]. Several studies have also reported about the production of cell wall-degrading enzymes, such as chitinase, β-1,3-glucanase, and protease, from various PGPBs that could potentially control plant fungal disease by antagonizing the growth and infection of phytopathogens [28,29,30,31,32,34]. These cell wall-degrading enzymes degrade the cell wall components of phytopathogens, which are mainly composed of chitin, glucan, and glycoprotein matrix [34,35,36]. Cell wall-degrading enzymes from PGPB have been reported to exhibit a wide range of antagonistic abilities against plant pathogens, such as *Colletotrichum gloeosporioides*, *Fusarium oxysporum*, *Pestalotia diospyri*, *Botrytis cinerea*, and *Botryosphaeria dothidea* [28,29,30,31,32].

In addition, PGPBs have been widely reported to enhance plant growth and productivity [29,30,34,37]. PGPBs increase plant growth through the secretion of plant growth hormones and increase the availability and uptake of plant nutrients [38,39,40,41,42,43]. Specifically, PGPB produce auxins, such as indole-3-acetic acid (IAA), which induces the growth of root hairs and lateral roots in plants for enhanced nutrient uptake [31,32,34,39,41]. For nutrient availability, PGPBs have been reported to enhance the nitrogen content in plants through various processes including ammonia production, nitrogen fixation, and nitrogen remobilization [38,41,42]. PGPB also increases phosphate availability in the soil through the solubilization of inorganic phosphate, which could potentially enhance root density and further promote nutrient absorption and plant growth [40,43,44]. Moreover, the enhancement of soil nitrogen content and uptake by plants also increases the chlorophyll content in leaves, which in turn enhances the photosynthetic activity and biomass production [45,46,47,48]. Despite the various reports about the use of PGPB in the production of forest seedlings, the potential for simultaneous control of leaf blight caused by *P.maculans* and growth promotion of *Q. acutissima* container seedlings using the *B. velezensis* strain has not been established. For successful reforestation of *Q. acutissima* trees, high-quality container seedlings with a well-developed root system and minimal root injuries should be used to avoid pathogen infections, and enhance successful resource competition and rapid growth after out planting [11,12,14,49]. Therefore, the objective of this study was to investigate the biocontrol potential of *B. velezensis* CE 100 against leaf blight disease caused by *P. maculans* and growth promotion of *Q. acutissima* container seedlings.

## 2. Results

### 2.1. Antagonistic Activity of Bacillus velezensis CE 100 against Pestalotiopsis maculans

#### 2.1.1. Cell Growth and Lytic Enzyme Production by *Bacillus velezensis* CE 100

**Figure 2:**
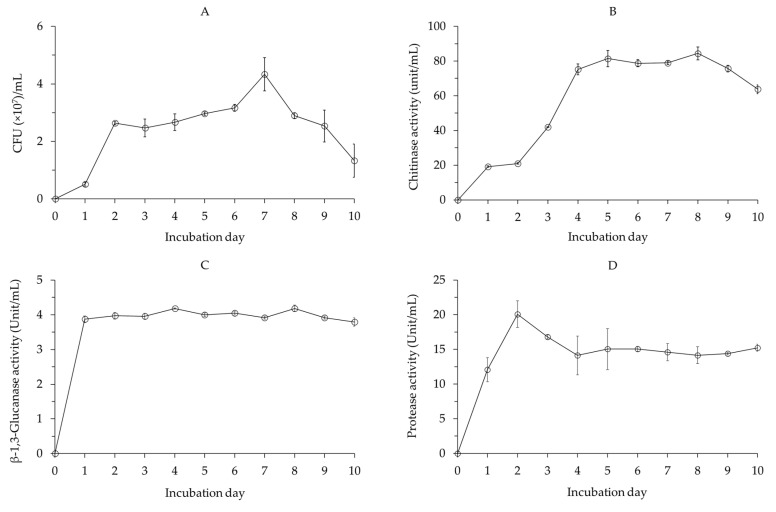
Cell growth curve (**A**), chitinase activity (**B**), β-l,3-glucanase activity (**C**), and protease activity (**D**) of *Bacillus velezensis* CE 100. Values are presented as means ± standard deviation (*n* = 3).

The cell growth of *B. velezensis* CE 100 increased rapidly until 2 days and then gradually increased between 2 and 7 days after inoculation (Figure 2A). The highest cell growth of 4.33 × 10^7^ colony-forming units (CFU)/mL was observed 7 days after inoculation. After 7 days, cell growth gradually decreased until the end of the experiment (Figure 2A).

The chitinase activity of *B. velezensis* CE 100 increased until 5 days after inoculation (Figure 2B). The activity of chitinase then slightly declined, before increasing again to the maximum value of 84.40 unit/mL after 8 days of inoculation. After 8 days, the activity of chitinase exhibited by *B. velezensis* CE 100 declined (Figure 2B).

The β-1,3-glucanase activity of *B. velezensis* CE 100 increased sharply 1 day after inoculation to 3.87 units/mL (Figure 2C). Then, *B. velezensis* CE 100 exhibited β-1,3-glucanase enzyme activity between the range of 3.79 and 4.18 units/mL until the end of the experiment (Figure 2C).

The protease activity of *B. velezensis* CE 100 increased to a maximum value of 20.08 units/mL 2 days after inoculation (Figure 2D). Then, the activity of protease declined to 14.13 units/mL by 4 days after inoculation and remained relatively constant (between 14.13 and 15.23 units/mL) until the end of the experiment (Figure 2D).

#### 2.1.2. Antagonistic Activity of *Bacillus velezensis* CE 100 against *Pestalotiopsis maculans*

**Figure 3:**
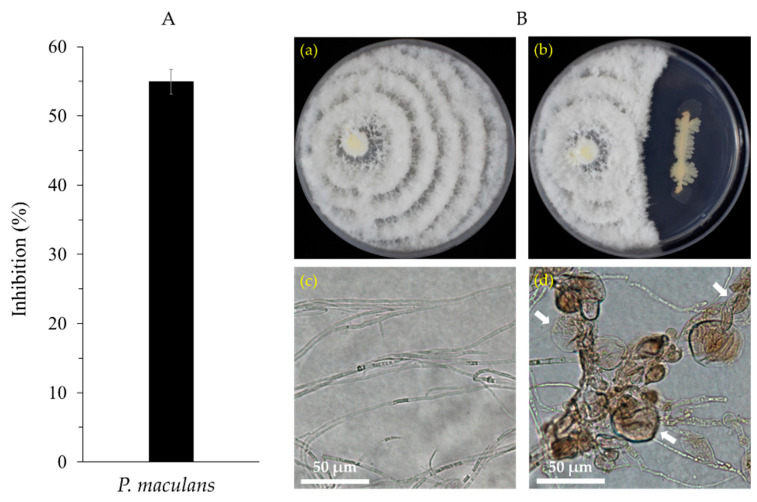
Growth inhibition percentage of *Pestalotiopsis maculans* by *Bacillus velezensis* CE 100 (**A**). Normal mycelial growth of *P. maculans* (**B**(**a**)) and antagonistic activity of *B. velezensis* CE 100 against mycelial growth of *P. maculans* by the dual culture method (**B**(**b**)). Normal hyphal morphology of *P. maculans* from the control (**B**(**c**)) and deformed hyphae of *P. maculans* from dual culture with *B. velezensis* CE 100 (**B**(**d**)), observed under a light microscope (200× magnification). Error bar represents the standard deviation (*n* = 3). Arrows indicate hyphal deformation caused by *B. velezensis* CE 100.

*B. velezensis* CE 100 inhibited the mycelial growth of *P. maculans* by 54.94% (Figure 3A). The microscopic observation revealed that treatment with *B. velezensis* CE 100 caused abnormal hyphae morphology characterized by swelling, degradation, and deformation (Figure 3B(d)), compared to normal hyphae morphology in the control (Figure 3B(c)).

#### 2.1.3. Survival Rate of *Quercus acutissima* Seedlings

**Figure 4:**
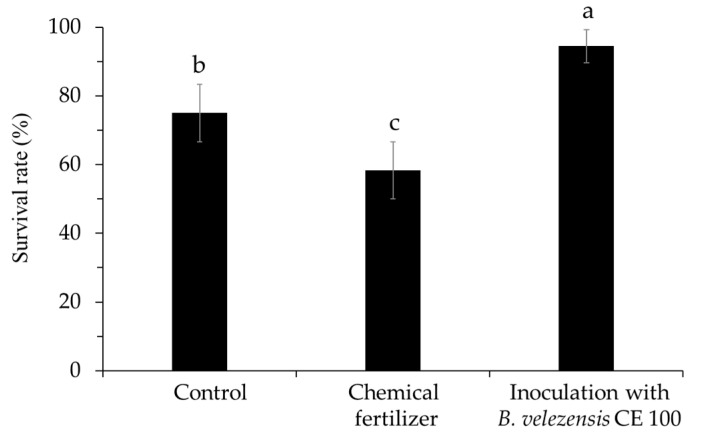
Survival rate of *Quercus acutissima* seedlings in different experimental groups. Values are presented as means ± standard deviation (*n* = 3). Different superscripts a, b and c indicate significantly different values according to the least significant difference test (LSD), *p* = 0.003.

Leaf blight disease caused by *P. maculans* decreased the survival rate of oak seedlings across all treatment groups (Figure 4). However, the survival rate of seedlings inoculated with *B. velezensis* CE 100 was significantly higher compared to seedlings in the chemical treatment and the control. Inoculation with *B. velezensis* CE 100 increased the survival rate of *Q. acutissima* seedlings by 1.6-fold and 1.3-fold compared to the chemical treatment and control, respectively (Figure 4). Moreover, the survival rate of seedlings in the chemical fertilizer treatment was significantly lower compared to the control (Figure 4).

### 2.2. Growth Promotion Effect of Bacillus velezensis CE 100 on Quercus acutissima Seedlings

#### 2.2.1. Indole-3-Acetic Acid (IAA) Production

**Figure 5:**
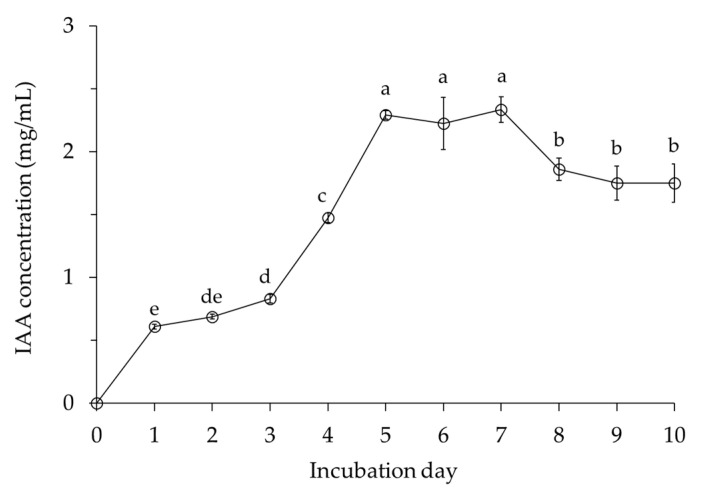
Indole-3-acetic acid (IAA) production by *Bacillus velezensis* CE 100. Values are presented as means ± standard deviation (*n* = 3). Different superscripts a–e indicate significantly different values according to the least significant difference test (LSD), *p* < 0.001.

IAA gradually increased until 3 days after inoculation and then sharply increased to 2.2 mg/mL at 5 days (Figure 5). *B. velezensis* CE 100 maintained high IAA production, with a range of 2.22 to 2.33 mg/mL between 5 and 7 days after inoculation, followed by a slight decrease until the end of the study. The highest level of IAA production was consistently the period of highest cell growth.

#### 2.2.2. Nutrient Contents in the Growth Media

The total nitrogen content in the growth media of *Q. acutissima* container seedlings inoculated with *B. velezensis* CE 100 (2.85 g/kg) or chemical fertilizer treatment (2.98 g/kg) was significantly higher compared to the control (2.63 g/kg) (Table 1). Similarly, the total phosphorus content in the growth media of *Q. acutissima* seedlings inoculated with *B. velezensis* CE 100 (0.44 g/kg) or chemical fertilizer treatment (0.57 g/kg) was significantly higher compared to the control (0.19 g/kg). Thus, inoculation with *B. velezensis* CE 100 increased the total nitrogen and total phosphorus contents in the growth media by approximately 1.1-fold and 2.3-fold relative to the control, respectively. However, the chemical fertilizer showed a significantly higher total phosphorus content in the growth media compared to the *B. velezensis* CE 100 treatment (Table 1).

#### 2.2.3. Nutrient Concentration and Content of *Quercus acutissima* Seedlings

Inoculation with *B. velezensis* CE 100 increased the concentration of total nitrogen by 1.3-fold and 1.9-fold and total phosphorus by 1.6-fold and 2.2-fold in *Q. acutissima* seedlings compared to the chemical fertilizer treatment or control, respectively (Table 1).

The nutrient content (total nitrogen and total phosphorus) of *Q. acutissima* seedlings was significantly higher in the group inoculated with *B. velezensis* CE 100 (931.71 and 370.10 mg/plant) compared to the chemical fertilizer treatment (722.35 and 147.66 mg/plant) and the control group (303.47 and 79.0 mg/plant) (Table 1). Inoculation with *B. velezensis* CE 100 increased the total nitrogen content by 1.3-fold or 3.1-fold and the total phosphorus content by 2.5-fold or 4.7-fold in *Q. acutissima* seedlings compared to the chemical fertilizer treatment and control, respectively (Table 1).

#### 2.2.4. Biomass of *Quercus acutissima* Seedlings

The total biomass (shoots and roots) was highest in *Q. acutissima* seedlings inoculated with *B. velezensis* CE 100 (164.64 g/plant) followed by chemical fertilizer treatment (122.61 g/plant), while seedlings in the control had the lowest biomass (76.62 g/plant) (Table 2). Inoculation with *B. velezensis* CE 100 increased the shoot dry mass of *Q. acutissima* seedlings by 1.3-fold and 2.5-fold compared to the chemical fertilizer treatment and control, respectively. Similarly, the root dry mass increased by 1.4-fold and 1.7-fold in *Q. acutissima* seedlings inoculated with *B. velezensis* CE 100 compared to the chemical fertilizer treatment and control, respectively. The total biomass of seedlings inoculated with *B. velezensis* CE 100 was 1.3-fold and 2.1-fold higher compared to the seedlings under the chemical fertilizer treatment and control, respectively (Table 2).

#### 2.2.5. Chlorophyll Index of *Quercus acutissima* Seedlings

**Figure 6:**
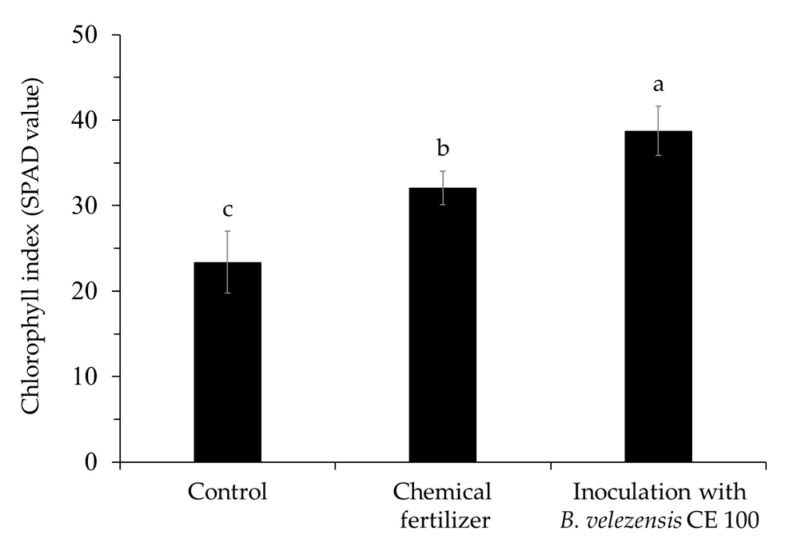
Chlorophyll index of *Quercus acutissima* seedlings in each treatment group: control, chemical fertilizer treatment, and inoculation with *Bacillus velezensis* CE 100. Error bars represent the standard deviation (*n* = 9). Different superscripts a, b and c indicate significantly different values according to the least significant difference test (LSD), *p* < 0.001.

Inoculation with *B. velezensis* CE 100 significantly increased the chlorophyll index in the leaves of *Q. acutissima* seedlings, compared to the chemical fertilizer treatment and the control group (Figure 6). The chlorophyll index of seedlings treated with *B. velezensis* CE 100 (38.74 SPAD value) was 1.2-fold and 1.7-fold higher than the content in seedlings under the chemical fertilizer treatment (32.08 SPAD value) and the control (23.36 SPAD value), respectively.

## 3. Discussion

### 3.1. Antagonistic Activity of Bacillus velezensis CE 100 against Pestalotiopsis maculans

Phytopathogenic fungal cell walls, which are mainly composed of chitin, glucans, and glycoproteins polymers, are vital in maintaining structural integrity and metabolic activities of fungal cells, which affects their survival, growth, and pathogenicity [50,51]. Thus, cell wall-degrading enzymes, such as chitinase, β-l,3-glucanase, and protease enzyme produced by PGPB, have potential to cause alterations in the structural and functional properties of fungal cell walls, which ultimately inhibits mycelial growth, sporulation, and plant infection by phytopathogenic fungi [50,51,52]. In this study, *B. velezensis* CE 100 produced chitinase, β-l,3-glucanase, and protease enzymes (Figure 2B–D) and exhibited a strong antifungal effect against *P. maculans* (Figure 3)*,* the causal agent of leaf blight disease in *Q. acutissima*. The mycelium of *P. maculans* grown in dual culture with *B. velezensis* CE 100 showed abnormal hyphae morphology, such as swelling, degradation, and deformation, when observed under a light microscope (Figure 3B(d)). The reduced stiffness and degradation of the fungal cell wall caused by chitinase, β-l,3-glucanase and protease enzymes exposes the cell membrane to low osmotic pressure environments and external aggressors, such as environmental fluxes and plant defense chemicals, which inhibit growth, leading to transformation and cell death [28,51]. These results are consistent with previous studies that reported the production of cell wall-degrading enzymes by *Bacillus licheniformis* MH48, *B. velezensis* strain HYEB5-6, and *B. velezensis* CE 100, which hydrolyses the fungal cell wall components, and results in cell wall degradation, deformation, and lysis of phytopathogenic fungi, such as *P. diospyri*, *Pestalotiopsis karstenii*, *B. cinerea*, and *C. gloeosporioides* [28,30,52]. Therefore, chitinase, β-l,3-glucanase, and protease production by *B. velezensis* CE 100 could potentially cause cell wall disintegration and mycelial deformation due to the lysis of chitin, glucan, and glycoprotein polymers in the phytopathogenic fungal cell wall [50]. This potentially led to mycelial growth inhibition of *P. maculans*, which consequently lowered the pathogen virulence and thus reduced the rate of infection [51].

In this study, inoculation with *B. velezensis* CE 100 increased the survival rates of *Q. acutissima* seedlings by 1.6-fold and 1.3-fold compared to the chemical fertilizer treatment and control, respectively (Figure 4). The lowest survival rate of seedlings from leaf blight disease caused by *P. maculans* was observed in the chemical fertilizer treatment (Figure 4), which could be attributed to the effect of chemical nitrogen fertilization on the promotion of phytopathogenic fungal density [16]. In this study, the nitrogen content in the growth media was significantly higher in both the chemical fertilizer treatment and *B. velezensis* CE 100 inoculation compared to the control (Table 1). High nitrogen accumulation in soil from chemical nitrogen fertilization could potentially reduce the diversity of the soil microbial communities in a manner that negatively effects plant health due to a substantial increase in the proportion of phytopathogenic fungi [16]. Even though the nitrogen content in the growth media was not significantly different between the chemical fertilizer treatment and *B. velezensis* CE 100 inoculation, the survival rate of *Q. acutissima* seedlings was significantly higher in *B. velezensis* CE 100 (Figure 4). This is due to the cell wall-degrading activity of chitinase, β-l,3-glucanase, and protease produced by *B. velezensis* CE 100, which reduced the growth and infection rate of *P. maculans* fungus. Thus, inoculation with *B. velezensis* CE 100 reduced leaf blight disease incidence and improved the survival rate of *Q. acutissima* seedlings compared to the chemical fertilizer treatment (Figure 4). These results suggest that *B. velezensis* CE 100 can play an effective role as a biocontrol agent against *Pestalotiopsis* blight in the production of healthy *Q. acutissima* container seedlings in forest nurseries.

### 3.2. Growth Promotion of Quercus acutissima Container Seedlings by Bacillus velezensis CE 100

PGPB are known to promote plant growth via the secretion of phytohormones, such as indole-3-acetic acid (IAA), which induces division, expansion, and differentiation of plant cells [34,39,41]. IAA also stimulates lateral root and root hair growth and development [34,39,41]. A well-developed root system due to the activity of IAA increases the surface area for nutrient absorption from the soil, which in turn enhances plant growth and biomass production [34,39,41]. In addition, PGPB promotes plant growth by enhancing the availability and uptake of essential plant nutrients, such as nitrogen and phosphorus [38,40,42,43]. PGPB can promote plant growth and development by increasing the uptake of nitrogen, which is assimilated with other nutrients, such as carbon, hydrogen, oxygen, and sulfur, to produce amino acids necessary for protoplasm formation [53]. Additionally, besides the formation of protoplasm, which is essential for cell division, nitrogen is an integral component of plant proteins (including enzymes), vitamins, and dry mass, which makes it the main basis for plant growth and development [53]. PGPB also increases the availability and uptake of phosphorus, which is a major plant nutrient that stimulates root growth and thus, could potentially affect the uptake of other plant nutrients [40,43,44]. In this study, *B. velezensis* CE 100 produced IAA during cell growth (Figure 5), which could have increased the root biomass of *Q. acutissima* seedlings (Table 2). Due to the enhanced root development, seedlings inoculated with *B. velezensis* CE 100 had a higher total nitrogen and total phosphorus concentration and content compared to the chemical fertilizer treatment and control groups (Table 1). In addition, inoculation of *B. velezensis* CE 100 increased the total nitrogen and total phosphorus content in the growth media compared to the control (Table 1). PGPBs, such as *B. velezensis* CE 100, can increase the nitrogen availability in the growth media through ammonium production, nitrogen fixation, and nitrogen remobilization [38,42,52]. In addition, PGPB inoculation could have potentially increased the phosphorus content through phosphate solubilization [40,43,44]. Previously, Choub et al. [52] demonstrated the potential for ammonium production as well as phosphate solubilization by *B. velezensis* CE 100. Therefore, in this study, inoculation with *B. velezensis* CE 100 culture led to an increase in the nitrogen and phosphorus content in the growth media, which could have subsequently led to higher nutrient uptake by the roots. According to Radhapriya et al. [37], IAA produced by PGPB enhances the development of roots in forest seedlings, such as *Albizia lebbeck* (L.) Benth, *Gmelina arborea* Roxb, *Pongamia pinnata* (L.), and *Terminalia arjuna* (DC.) Wight & Arn and increases total biomass production. Thus, inoculation of *B. velezensis* CE 100 enhanced root development (Table 2) through IAA production and increased the availability of total nitrogen and total phosphorus contents in the growth media (Table 1). As a result, total nitrogen, and total phosphorus absorption by *Q. acutissima* seedlings was increased, which consequently improved seedling growth compared to the chemical fertilizer treatment and the control group (Table 2).

Moreover, the increase in the nitrogen content of *Q. acutissima* seedlings inoculated with *B. velezensis* CE 100 ultimately improved the chlorophyll index in the leaves (38.74 SPAD value), compared to the chemical fertilizer treatment (32.08 SPAD value) and the control (23.36 SPAD value) (Figure 6). Nitrogen is an important mineral that improves the synthesis of chlorophyll and thus enhances carbon assimilation by increasing the photosynthetic rate in plants [46,47,48]. Thus, increasing nitrogen uptake in plants is an important factor in promoting photosynthesis and hence, increasing plant growth [45,46,47,48]. In this study, the increase in the chlorophyll index in seedlings inoculated with *B. velezensis* CE 100 also corresponded with the observed increase in total biomass production by 1.3-fold and 2.2-fold compared to the chemical fertilizer treatment and control, respectively (Table 2). According to Salifu et al. [45], increasing the nitrogen content of *Quercus* (*Q. rubra*) seedlings improved the chlorophyll index and seedling biomass. Therefore, inoculation of *Q. acutissima* seedlings with *B. velezensis* CE 100 enhanced root development through IAA production, increased nitrogen uptake, and improved the leaf chlorophyll index and photosynthetic rate, which consequently increased biomass production compared to the chemical fertilizer treatment and control (Table 2). Hence, *B. velezensis* CE 100 can be effectively used as a biofertilizer to promote the growth of *Q. acutissima* container seedlings in forest nurseries.

## 4. Materials and Methods

### 4.1. Preparation and Growth Patterns of Bacillus velezensis CE 100

*B. velezensis* CE 100 was previously isolated from tomato pot soil [54]. Pure colonies of *B. velezensis* CE 100 were grown in sterile tryptone soy broth (TSB) at 30 °C for 3 days. The resultant culture broth (×10^7^ CFU/mL) was mixed with 50% glycerol and stored at −70 °C as original stock for future use. In this study, 10 μL of the original stock were spread on tryptone soy agar (TSA) medium and a single colony of *B. velezensis* CE 100 was selected and pre-inoculated in 50 mL of TSB medium and incubated at 120 rpm and 30 °C for 3 days using a H1012 Incu-Shaker (Benchmark Scientific, Inc., Edison, NJ, USA). The resultant culture (3 × 10^7^ CFU/mL) was then used in all the proceeding experiments as a pre-inoculation culture.

To observe the growth pattern, 1 mL/L of *B. velezensis* CE 100 culture from the pre-inoculation culture was inoculated in 0.5 L of pink broth (PB) media [34]. Briefly, PB media was composed of pink fertilizer 3 g/L, MgSO_4_ 7H_2_O 0.2 g/L, KH_2_PO_4_ 0.2 g/L, CaCO_3_ 0.1 g/L, NaCl 0.1 g/L, sucrose 3 g/L, chitin powder 0.5 g/L, yeast extract 0.6 g/L, and distilled water 0.5 L. The bacteria culture was incubated at 120 rpm and 30 °C for 10 days. During the incubation period, 1 mL of the bacterial culture was sampled daily to enumerate the colony-forming units, using the standard plate count method on tryptone soy agar (TSA) medium.

### 4.2. Isolation and Identification of Pestalotiopsis maculans

To isolate and identify the phytopathogenic fungi causing leaf blight in *Q. acutissima*, the leaves, twigs, and roots of the infected seedlings were cut into 1 cm with scissors sterilized with 70% ethanol. Each sample was sterilized in 70% ethanol for 5 min and then washed three times with sterile distilled water. The samples were air dried for 30 min in a clean bench and then inoculated onto PDA medium containing 0.05 g/L of streptomycin sulfate and cultured at 25 °C. The fungal growth from plant tissues was observed regularly for 14 days and unique colonies were hyphal-tipped onto new potato dextrose agar (PDA) plates according to their morphology, to obtain pure culture isolates. Molecular identification of the purely isolated fungi was done based on the 18S *ribosomal RNA* gene sequence from Macrogen Inc. (Seoul, Korea). The obtained gene sequences were compared with the sequences of other fungi deposited in the national center for biotechnology information (NCBI) database with a similarity of more than 98%, using the basic local alignment and search tool (BLAST, http://www.ncbi.nlm.nih.gov/BLASI (accessed on 29 September 2021)) program. A phylogenetic analysis based on the maximum likelihood method was done using the molecular evolutionary genetics analysis (MEGA 7, Temple University, Philadelphia, PA, USA). The identified strains were studied for pathogenicity based on leaf blight disease symptoms using healthy seedlings. *Q. acutissima* container seedlings had an average trunk diameter of 4.62 ± 1.39 mm and an average shoot height of 31.07 ± 8.74 cm at the time of infection. The causal agent of leaf blight in *Q. acutissima* seedlings was confirmed as *P. maculans*.

### 4.3. Lytic Enzyme Production by Bacillus velezensis CE 100

To measure the lytic enzyme activity, 1 mL/L of *B. velezensis* CE 100 culture from the pre-inoculation culture was inoculated into 0.5 L of PB medium and cultured at 30 °C and 120 rpm for 10 days. Then, 2 mL of bacterial culture broth of *B. velezensis* CE 100 were sampled daily for 10 days. The sampled *B. velezensis* CE 100 culture was centrifuged at 12,000 rpm for 10 min and the supernatants were used to analyze the lytic enzyme activity.

Analysis of the chitinase activity was performed according to Lingappa [55]. The reaction mixture was prepared by mixing 50 μL of *B. velezensis* CE 100 supernatant, 450 μL of 50 mM sodium acetate buffer (pH 5.0), and 500 μL of 0.5% colloidal chitin solution in a 2-mL tube and maintained at 37 °C for 1 h to complete the reaction. To stop the reaction, 200 μL of 1 N NaOH were added, and the solution centrifuged at 12,000 rpm and 4 °C for 7 min. Then, 750 μL of supernatant were mixed with 1 mL of Schales’ reagent (0.5 M sodium carbonate, 1.5 mM potassium ferricyanide) and 250 μL of deionized water and the mixture was boiled at 100 °C for 15 min. Then, the chitinase activity was measured based on the color development at 420 nm using a UV spectrometer (UV-1650PC, Shimadzu, Kyoto, Japan). One unit of chitinase activity was defined as the reducing activity that releases 1 µmol of N-acetyl-glucosamine per hour at 37 °C.

The β-l,3-glucanase activity was determined by measuring the glucose released from laminarin during a glucose oxidase reaction as previously described [34]. Briefly, the reaction mixture was prepared by mixing 0.4 mL of 50 mM sodium acetate (pH 5.0), 50 µL of 1% laminarin solution, and 50 μL of *B. velezensis* CE 100 supernatant and maintained at 37 °C for 1 h to complete the reaction. Then, 1.5mL of dinitro salicylic acid solution was added and the solution was boiled for 5 min to stop the reaction. The concentration of reducing sugars was measured at 550 nm using a UV spectrophotometer. One unit of β-1,3-glucanase activity was defined as the amount of enzyme that catalyzed the release of 1 µmol of glucose/mg of protein/hour.

Protease activity was determined as previously described [34]. Briefly, 100 mM of tris buffer containing 2 mM CaCl_2_ and 1% casein was prepared and adjusted to pH 8.0. A reaction mixture containing 50 µL of *B. velezensis* CE 100 supernatant and 950 µL of tris buffer was incubated at 60 °C for 15 min. Then, 500 µL of 20% trichloroacetic acid were added to terminate the reaction. The mixture was centrifuged for 15 min to remove the precipitate and the absorbance of the supernatant containing acid-soluble proteins was measured at 280 nm using a UV spectrophotometer. One unit of protease activity was defined as the amount of enzyme that liberated 1 µg of tyrosine per min.

### 4.4. Antagonistic Activity of Bacillus velezensis CE 100 against Pestalotiopsis maculans

The antagonistic activity of *B. velezensis* CE 100 against *P. maculans* isolated from infected *Q. acutissima* leaves was determined using the dual culture method [56], using PDA medium. Briefly, 10 µL of *B. velezensis* CE 100 culture from the pre-inoculation culture were sub-cultured to TSA medium at 30 °C for 24 h, while *P. maculans* was cultured in PDA medium at 25 °C for 7 days. To measure the growth inhibition, a 5-mm disk of *P. maculans* was made using a sterile cork-borer and placed on one side of the PDA plate and a loop of *B. velezensis* CE 100 culture was streaked on the other side of the same plate at a distance of 4 cm from the fungi. The control plates were inoculated with only *P. maculans*. All culture plates were incubated at 25 °C for 7 days and growth inhibition of *P. maculans* was measured by comparing the radial growth on dual culture plates with the radical growth on the control plates. Three replications were used, and all experiments were repeated three times. The mycelium growth inhibition rate was calculated using the following equation: Inhibition rate (%) = [(α − β)/α] × 100, where α is the radial growth length of *P. maculans* in the control and β is the radial growth length of the *P. maculans* of the dual culture plate [29,56].

Deformation of *P. maculans* mycelial morphology was observed from each treatment by selecting a small piece of mycelial growth on the border between the bacterial streak and the fungal growth. The mycelia were placed on a glass slide with a drop of water and covered with glass coverslips and examined under a light microscopic (BX41, Olympus, Tokyo, Japan) at 200× magnification.

### 4.5. Indole-3-Acetic Acid (IAA) Production by Bacillus velezensis CE 100

For quantitative analysis of IAA produced by *B. velezensis* CE 100, 1 mL/L of *B. velezensis* CE 100 culture from the pre-inoculation culture was inoculated into 0.5 L of PB medium and cultured at 30 °C and 120 rpm for 10 days. During the incubation period, 2 mL of the bacterial culture were sampled every day and the supernatant produced after centrifugation at 12,000 rpm was used for IAA analysis according to Salkowski’s method [57]. Briefly, 1 mL of *B. velezensis* CE 100 supernatant, 20 µL of orthophosphoric acid, and 2 mL of Salkowski reagent (35% perchloric acid 50 mL, 0.5 M FeCl_3_ solution 1 mL) were mixed. The mixture was reacted at room temperature under dark conditions for 30 min. Then, the IAA concentration of each sample was measured at 530 nm using a UV spectrometer.

### 4.6. Plant Material and Experimental Conditions

The seeds of *Q. acutissima* used in this study were collected from the 30-year-old *Q. acutissima* tree at College of Agriculture and Life Sciences in Chonnam National University, Korea (approximately 35°10′35.7″ N latitude, 126°54′08.2″ E longitude) in October 2016. Until use, it was stored in a refrigerator at a relative humidity of 40% and a temperature of 4 °C and surface sterilized by floating in 2.5% sodium hypochlorite solution (NaOCl) for 5 min, followed by five rinses with sterile distilled water before sowing.

The experiment was conducted at the forest nursery of Chonnam National University (approximately 35°10′23.2″ N latitude, 126°54′01.4″ E longitude) with three repetitions (Figure 1). Seeds were sown at a depth of 5 cm in a potted soil mixture of soil, sand, bed soil, and vermiculite at a ratio of 2:2:2:1. Seedling trays 25 cm wide, 41 cm long, and 16 cm high and containing 15 cells with a diameter of 7.5 cm and a height of 16 cm were used. After sowing in March 2017, until February 2018, only normal management was conducted and no treatment with fertilizers or bacterial inoculation done. Before fertilizer treatment or inoculation of microorganisms, the seedlings were transplanted into separate pots with a diameter of 20 cm and a height of 19 cm and the soil mixture was used in the same ratio as described above.

The average temperature range of the forest nursery was maintained between 5 and 27 °C. To prevent heat shock, when the outside temperature was higher than 27 °C, both sides of the forest nursery were opened, and a ventilation system was used to lower the temperature. The seedlings were grown under natural light conditions and the seedlings were watered once a week from March and April 2018 and November 2018 to April 2019 and once every other day from May to October 2018 and May to August 2019 using a watering can. The experiment was conducted in three treatments: control (only water with no fertilizer and bacterial inoculation), chemical fertilizer treatment, and inoculation with *B. velezensis* CE 100 treatment. Each treatment group contained 36 seedlings (three repetitions of 12 seedlings) and a total of 108 seedlings were used in the experiment. *B. velezensis* CE 100 inoculum was cultured at 30 °C for 7 days and applied on leaves and rhizosphere at a rate of 50 mL/plant. Bacterial inoculation was done at an interval of 10 days for effective control of fungal pathogens and to enhance plant growth as previously described [33,52]. For chemical fertilizer, 3 g/L of pink fertilizer (NPK 20-20-20) was applied to soils at a rate of 50 mL/plant at an interval of 10 days. Control seedlings received 100 mL of water per seedling every 10 days, without any bacteria or chemical fertilizer. All treatments were applied from March 2018 to August 2019.

### 4.7. Growth Medium, Plant Sampling, and Measurement

In August 2019, samples of growth media for nutrient analysis were collected from each pot and oven dried in a convection drying oven (VS-1202D4, Vision Scientific, Daejeon, Korea) at 65 °C for 24 h. The dry samples for each replication (*n* = 3) were homogeneously mixed and sieved through 2-mm wire mesh and maintained at room temperature for total nitrogen and total phosphorus analysis. To analyze the nutrient content of *Q. acutissima* seedlings, the seedlings were carefully removed from the growth media and the roots were washed in flowing tap water to remove all the debris. Then, seedlings were dried in a convection drying oven at 65 °C for 24 h to determine the dry weight. Each sample was pulverized and passed through a 30-mesh screen. Samples of at least nine seedlings in each replication were homogenously mixed and the homogenous samples for each replication (*n* = 3) were used for total nitrogen and total phosphorus analysis.

The total nitrogen content of growth media was analyzed using the Kjeldahl method after wet digestion with H_2_SO_4_ [58]. The total nitrogen content of seedlings was measured by the elemental analyzer (Variomax CN Analyzer, Elementar Analysensysteme GmbH, Langenselbold, Germany)) equipped with a thermal conductivity detector (TCD) after combustion at a high temperature (1200 °C) with nitrogen and helium gas. Total phosphorus of growth media and seedlings was analyzed using inductively coupled plasma optical emission spectrometry (Optima 8300, PerkinElmer, Waltham, MA, USA) after nitric acid decomposition using a microwave oven (MARS Xpress, CEM Co., Matthews, NC, USA). The analysis of total nitrogen and total phosphorus in both the growth media and seedlings was repeated three times for consistent results. The total nitrogen and total phosphorus content of the seedlings was calculated as described previously [30], using the following equation: Nutrient content (mg/plant) = [dry mass (g/plant) × nutrient concentration (%/plant)] × 10.

### 4.8. Chlorophyll Index of Quercus acutissima Seedlings

We analyzed the chlorophyll content in the seedlings according to Salifu et al. [45], which presents the SPAD values as the chlorophyll index. Briefly, the chlorophyll index in the leaves was measured by selecting nine seedlings showing relatively uniform growth in each replication. Nine leaves from the center of each seedling were randomly measured using a chlorophyll meter (SPAD-502 Plus, Konica Minolta, Tokyo, Japan). Five readings were taken from each leaf for a total of 45 measurements per plant. The measurements of the chlorophyll index were taken on the same day before sampling the seedling in August 2019.

### 4.9. Survival Rate of Quercus acutissima Seedlings

The survival rate of seedlings was investigated in August 2019. When the leaves of the seedlings were all dried, it was considered to be died. The survival rate of seedlings was calculated as a percentage of dead seedlings to the total number of seedlings in each replicate.

### 4.10. Statistical Analysis

Data were subjected to analysis of variance (ANOVA) using the SPSS (Statistical Package for the Social Sciences) statistical program (SPSS 25.0, SPSS Inc., Chicago, IL, USA). The mean values were compared by least significant difference (LSD) at *p* = 0.05.

## 5. Conclusions

Effective control of fungal diseases and nutrient management are essential in the production of high-quality forest container seedlings that are required for successful afforestation. *B. velezensis* CE 100 produced cell wall-degrading enzymes, such as chitinase, β-l,3-glucanase, and protease, and exhibited potential to inhibit the growth of *P. maculans*, the causal agent of leaf blight in *Q. acutissima* seedlings. Consequently, inoculation of *B. velezensis* CE 100 improved the survival rate of *Q. acutissima* seedlings in the forest nursery compared to the chemical fertilizer treatment and the control group. In addition, *B. velezensis* CE 100 produced IAA and enhanced the root growth of *Q. acutissima* seedlings, which improved the nutrient uptake and photosynthetic rate by increasing the chlorophyll content in the leaves, compared to the chemical fertilizer treatment and the control group. As a result, inoculation of *B. velezensis* CE 100 significantly enhanced the growth and biomass production of *Q. acutissima* container seedling. These results demonstrate that the use of eco-friendly *B. velezensis* CE 100 as an alternative to the use of chemicals not only plays an effective role in the biocontrol control of fungal diseases but also enhances the growth of high-quality container seedlings for successful afforestation programs.

## Figures and Tables

**Table 1:** Total nitrogen and total phosphorus in the growth media and in *Quercus acutissima* seedlings for each experimental group: control, chemical fertilizer treatment, and inoculation with *Bacillus velezensis* CE 100.

Treatment	Growth Media		Seedling			
	Nutrient Content (g/kg)		Nutrient Concentration (%/plant)		Nutrient Content (mg/plant)	
	Total Nitrogen	Total Phosphorus	Total Nitrogen	Total Phosphorus	Total Nitrogen	Total Phosphorus
Control	2.63 ± 0.14 ^b^	0.19 ± 0.01 ^c^	0.39 ± 0.08 ^c^	0.10 ± 0.01 ^c^	303.47 ± 45.98 ^c^	79.00 ± 1.02 ^c^
Chemical fertilizer	2.98 ± 0.08 ^a^	0.57 ± 0.09 ^a^	0.59 ± 0.06 ^b^	0.14 ± 0.02 ^b^	722.35 ± 117.59 ^b^	147.66 ± 1.01 ^b^
Bacterial inoculation	2.85 ± 0.16 ^a^	0.44 ± 0.05 ^b^	0.74 ± 0.03 ^a^	0.22 ± 0.04 ^a^	931.71 ± 54.96 ^a^	370.10 ± 2.76 ^a^

Means values are means ± standard deviation (*n* = 3). Different superscripts a, b and c within each column indicate significantly different values according to the least significant difference test (LSD), *p* < 0.001.

**Table 2:** Biomass production of *Quercus acutissima* seedlings in each treatment group: control, chemical fertilizer treatment, and inoculation with *Bacillus velezensis* CE 100.

Treatment	Shoot Dry Mass (g/plant)	Root Dry Mass (g/plant)	Total Dry Mass (g/plant)
Control	35.76 ± 6.45 ^c^	43.85 ± 7.63 ^c^	76.62 ± 13.69 ^c^
Chemical fertilizer	68.09 ± 6.95 ^b^	54.52 ± 12.05 ^b^	122.61 ± 13.67 ^b^
Bacterial inoculation	88.22 ± 8.79 ^a^	76.42 ± 13.57 ^a^	164.64 ± 16.24 ^a^

Means values are means ± standard deviation (*n* = 12). Different superscripts a, b and c within each column indicate significantly different values according to the least significant difference test (LSD), *p* < 0.001.

## Data Availability

All the data relevant to this manuscript is available on request from the corresponding author.
